# A Community-Adapted Approach to SARS-CoV-2 Testing for Medically Underserved Populations, Rhode Island, USA

**DOI:** 10.3201/eid2709.204874

**Published:** 2021-09

**Authors:** Matthew Murphy, Imshan Dhrolia, Alexandra Zanowick-Marr, Jun Tao, Cassie Sutten Coats, Siena Napoleon, Yelena Malyuta, Emily Adams, Trisha Arnold, Philip A. Chan, Amy Nunn

**Affiliations:** The Rhode Island Public Health Institute, Providence, Rhode Island, USA (M. Murphy, C. Sutten Coats, Y. Malyuta, E. Adams, T. Arnold, P.A. Chan, A. Nunn);; Brown University, Providence (M. Murphy, I. Dhrolia, J. Tao, P.A. Chan, A. Nunn);; The Miriam Hospital, Providence (A. Zanowick-Marr, S. Napoleon, P.A. Chan)

**Keywords:** COVID-19, coronavirus disease, SARS-CoV-2, severe acute respiratory syndrome coronavirus 2, viruses, respiratory infections, zoonoses, testing, vulnerable populations, Rhode Island, United States

## Abstract

We developed a testing program for severe acute respiratory syndrome coronavirus 2 in an urban Latinx neighborhood in Providence, Rhode Island, USA. Approximately 11% of Latinx participants (n = 180) tested positive. Culturally tailored, community-based programs that reduce barriers to testing help identify persons at highest risk for coronavirus disease.

As of May 2021, severe acute respiratory syndrome coronavirus 2 (SARS-CoV-2) had infected >154 million globally and caused >3.2 million deaths (*1*). The United States accounts for ≈21% of coronavirus disease (COVID-19) cases and related deaths worldwide (*1*). Vaccines are a highly effective transmission prevention tool. However, SARS-CoV-2 testing, contact tracing, and quarantining are among the few effective prevention measures available to the public that are proven to reduce transmission in the setting of variable vaccine availability and uptake (*2*). The COVID-19 disease burden has disproportionately affected Black and Latinx populations in the United States (*3*,*4*). These health disparities in racial and ethnic minority populations are driven by complex social and structural factors, such as a paucity of health services, absence of culturally tailored services, and economic barriers that affect adherence to quarantine guidelines (*5*,*6*). These disparities have been further compounded by fragmented SARS-CoV-2 testing policies in the United States, which have not prioritized testing for medically underserved racial and ethnic minority communities (*2*).

Rhode Island experienced high rates of SARS-CoV-2 infection early in the pandemic and has been recognized for expanding testing early across the state (*5*). Policies in Rhode Island evolved in tandem with the pandemic and availability of testing supplies. In April 2020, faced with limited testing supplies and healthcare personnel, officials in Rhode Island restricted SARS-CoV-2 testing to prescheduled appointments for symptomatic persons with recent travel histories (*7*,*8*). By June, testing recommendations in Rhode Island had evolved to include populations considered at high risk for COVID-19, and the state has since maintained one of the highest per capita testing rates in the United States (*7*,*8*). However, most testing locations required appointments and were limited to symptomatic patients, and services were not offered in most urban communities, where infection rates were highest. 

As seen elsewhere in the United States, the Latinx community in Rhode Island has been disproportionately affected by COVID-19 (*9*). The Latinx community constitutes just 14% of the population in Rhode Island; the most represented countries and territories are Mexico (35%), Cuba (29%), Spain (11.7%), and Puerto Rico (8.9%). However, the Latinx community accounts for 38% of positive SARS-CoV-2 tests and 33% of COVID-19–related hospitalizations in the state (*1*,*8*).

## The Study

In June 2020, to respond to the unmet need for culturally tailored SARS-CoV-2 testing services, we opened a multilingual, community-based site for testing by PCR in Providence, Rhode Island (*7*). This program was supported by the Rhode Island Department of Health. We partnered with a local community cultural center to develop a culturally tailored model for SARS-CoV-2 testing in urban neighborhoods with large numbers of Latinx residents and high rates of COVID-19 infection. The cultural center was a well-known space for community gatherings, artist performances, and religious services. The testing site was staffed by trained medical personnel including clinical providers and volunteers. We designed the testing model to accept all walk-ins; offer drive-through and walk-up testing; provide onsite access to bilingual testing services in English, Spanish, and Portuguese (with additional language services provided by tele-interpretation); offer patients multiple modalities for accessing test results (in person, by telephone, postal mail, or online portal for patients with email addresses); provide testing regardless of insurance status, provider referral, in-state residency, or clinical manifestation; and forego out-of-pocket costs. We also worked with Latinx community leaders to promote our testing program on Latinx radio, Facebook, and other social media; conducted outreach to sexual and gender minority communities on social media platforms; and partnered with established community resources (e.g., cultural centers, churches) to promote testing by word of mouth. All persons who underwent testing were required to provide their legal name and date of birth, proof of identity and address (e.g., state identification card, utility bill, bank statement, etc.), contact information (i.e., address, phone number), and insurance information if applicable. We did not collect information related to immigration or in-state residency status to avoid introducing perceived barriers to testing. A healthcare provider at our facility contacted every person who tested positive for SARS-CoV-2 as soon as results were available. Patients were then connected with available support services, such as food and housing resources. The Department of Health also contacted patients to support additional transmission prevention activities.

During June 8–August 8, 2020, a total of 498 persons in the community underwent testing at this site; 40% of the sample identified as Latinx. Approximately ≈5% of all persons (Table 1) and 11% of Latinx participants were SARS-CoV-2–positive, compared with a statewide positive rate of 2%–3% (*10*). Furthermore, although 40% of the sample self-identified as Latinx, Latinx persons constituted 80% of positive case-patients. Latinx persons had 7 times higher odds of testing positive (crude odds ratio [OR] 7.03, 95% CI 2.58–19.19) than did non-Latinx persons (Table 2). 

**Table 1:** Demographic information for patients undergoing testing for severe acute respiratory syndrome coronavirus 2 at Rhode Island Public Health Institute testing site, Rhode Island, USA, June 8–August 8, 2020*

Characteristic	Total, n = 498	SARS-CoV-2–positive patients, n = 26 (5%)		SARS-CoV-2–negative patients, n = 472 (95%)		
Median age (range), y	36.9 (7–91)	40.1 (7–91)		36.7 (11–77)		
Age group, y						
0–14	13 (3)	1 (4)		12 (3)		
15–34	262 (53)	11 (42)		251 (53)		
35–64	184 (37)	11 (42)		173 (37)		
>65	39 (8)	3 (12)		36 (8)		
Race						
White	230 (49)	2 (9)		228 (51)		
Other race	123 (26)	14 (61)		109 (24)		
Black or African American	86 (18)	4 (17)		82 (18)		
Asian	26 (6)	2 (9)		24 (5)		
American Indian or Alaskan Native	4 (1)	1 (4)		3 (1)		
Unknown or not reported	29 (6)	3 (12)		26 (6)		
Ethnicity						
Not Hispanic or Latinx	303 (63)	5 (21)		298 (65)		
Hispanic or Latinx	180 (37)	19 (79)		161 (35)		
Unknown or not reported	15 (3)	2 (8)		13 (3)		
Preferred language						
English	392 (79)	12 (48)		380 (81)		
Spanish	102 (21)	12 (48)		90 (19)		
Other language	2 (0)	1 (4)		1 (0)		
Unknown or not reported	2 (0)	1 (4)		1 (0)		
Sex assigned at birth						
F	304 (61)	9 (36)		295 (63)		
M	193 (39)	16 (64)		177 (38)		
Unknown or not reported	1 (0)	1 (4)		0		
Gender identity (grouped)						
Woman	225 (52)	9 (41)		216 (53)		
Man	155 (36)	13 (59)		142 (35)		
Nonbinary, genderqueer, nonconforming, or agender	46 (11)	0		46 (11)		
Not listed or other	5 (1)	0		5 (1)		
Unknown or not reported	67 (13)	4 (15)		63 (13)		
Sexual orientation						
Straight or heterosexual	260 (60)	20 (91)		240 (59)		
Pansexual, queer, asexual, or bisexual	100 (23)	0		100 (24)		
Lesbian, gay, or homosexual	57 (13)	1 (5)		56 (14)		
Not listed or other	15 (3)	1 (5)		14 (3)		
Unknown or not reported	66 (13)	4 (15)		62 (13)		
Clinical manifestation						
Asymptomatic	369 (90)	14 (78)		355 (91)		
Symptomatic	39 (10)	4 (22)		35 (9)		
Unknown or not reported	90 (18)	8 (31)		82 (17)		
Insurance package						
Commercial or group policy, e.g., HMO, PPO	307 (62)	10 (40)		297 (63)		
No insurance	116 (23)	10 (40)		106 (23)		
Medicaid or Medicare Part B	73 (15)	5 (20)		68 (14)		
Unknown or not reported	2 (0)	1 (4)		1 (0)		

*Values are no. (%) except as indicated. SARS-CoV-2, severe acute respiratory syndrome coronavirus 2.

**Table 2:** Association between specific sociodemographic characteristics and a positive PCR test result for severe acute respiratory syndrome coronavirus 2, Rhode Island, USA, June 8–August 8, 2020*

Variables	Crude odds ratio (95% CI)	Adjusted odds ratio (95% CI)
Age, y	1.01 (0.99–1.03)	NC
Race		
White	Referent	
Asian	9.5 (1.28–70.52)	NC
Black/African American	5.56 (1.00–30.93)	NC
Other Race	15.27 (3.43–67.92)	NC
Ethnicity		
Non-Hispanic	Referent	
Hispanic	7.03 (2.58–19.19)	NC
Gender		
Female	Referent	
Male	2.96 (1.28–6.84)	NC
Insurance status		
Insured		Referent
Uninsured	NC	1.46 (0.50–4.21)
Medicaid/Medicare Part B	NC	2.57 (0.75–8.75)
Sexual orientation		
Heterosexual		Referent
Same-sex	NC	0.61 (0.07–5.47)
Bisexual	NC	NC
Queer, asexual, or pansexual	NC	NC
Other	NC	0.69 (0.08–5.97)

*In an exploratory analysis, we treated demographics, insurance, and sexual orientations as exposures and identified models for each variable. Because no factors could affect the status of age, race, or ethnicity, we present crude odds ratios for these variables. For insurance and sexual orientation, we identified age, race, and ethnicity as confounding variables on the basis of a priori knowledge and present adjusted odds ratios. NC, not calculable.

**Figure 1:**
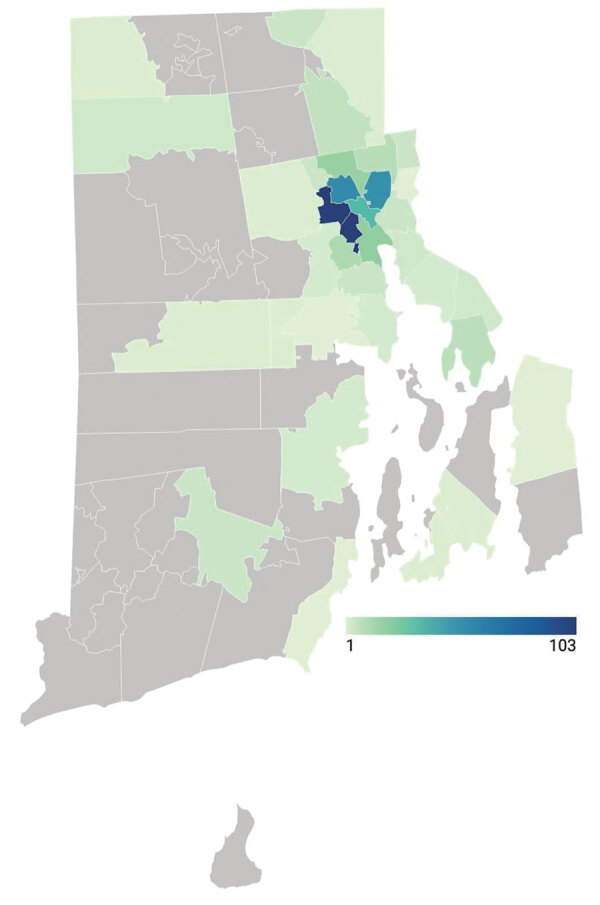
Geographic distribution of 498 persons tested for severe acute respiratory syndrome coronavirus 2 by Rhode Island Public Health Institute staff, Rhode Island, USA, June 8–August 8, 2020. Color scale indicates number of persons tested by ZIP code. Five patients had unknown ZIP codes and 16 were from out of state.

**Figure 2:**
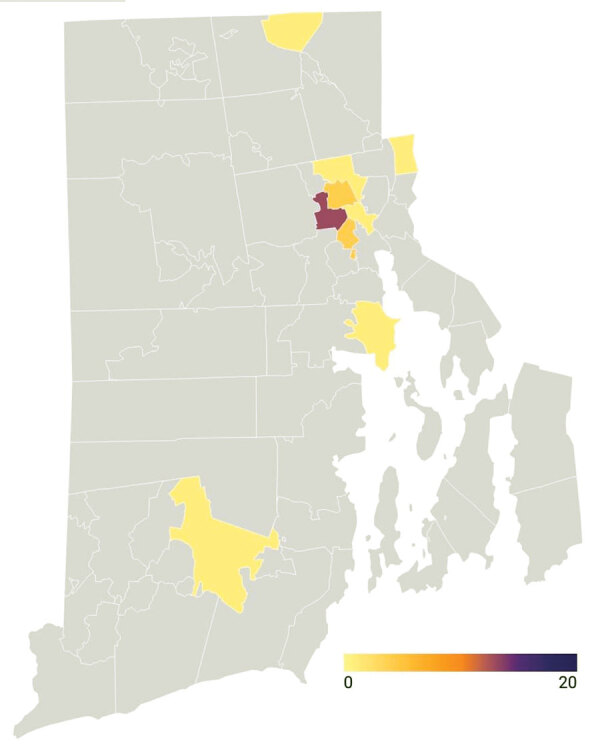
Geographic distribution of 28 persons receiving a positive test result for severe acute respiratory syndrome coronavirus 2 from testing performed by Rhode Island Public Health Institute staff during June 8–August 8, 2020. Color scale indicates number of persons testing positive by ZIP code. One patient had an unknown ZIP code.

Although we designed our program to respond to unmet need for urban SARS-CoV-2 testing, it attracted persons from throughout the city of Providence and the state of Rhode Island (Figure 1). However, the greatest number of positive SARS-CoV-2 tests were from persons who lived in the surrounding ZIP codes (Figure 2), a geographic area experiencing high rates of community transmission.

Only 39% of all patients in this sample were men, but they represented 59% of all COVID-19 cases. Being male was associated with 2.96 times higher odds of testing positive (crude OR 2.96, 95% CI 1.28–6.84). Sexual minorities accounted for ≈40% of the sample, and gender minorities accounted for 12% of the sample. However, sexual and gender minorities had far lower rates of COVID-19 infection; 90% of persons who tested positive for SARS-CoV-2 were cisgender and heterosexual.

## Conclusions

Our experience suggests that SARS-CoV-2 testing models that reduce barriers to care can successfully reach medically underserved communities that have high rates of COVID-19 infection. Culturally tailored approaches might be critical for identifying Latinx populations unaware of their SARS-CoV-2 infection (*10*). Not requiring health insurance or physician orders for testing, not charging payment, and offering walk-up and drive-through testing enabled widespread participation in our testing program. Offering multiple means of bilingual communication, including text, phone, email, traditional mail, and an online portal, also enabled communication with otherwise hard-to-reach patients. Although our findings are notable, they would be strengthened by an increased sample size to better characterize differences observed in the study population. Our outreach strategies were effective, but additional efforts in future initiatives could further improve testing outreach.

Our program provides a useful framework for reducing barriers to SARS-CoV-2 testing services in underserved communities, including sexual and gender minorities and Latinx populations who otherwise might not be tested for SARS-CoV-2. Perhaps the greatest challenge to replicating and sustaining this model is developing a viable funding model. Despite our program’s success in enabling testing for persons at elevated risk for COVID-19, the human and financial resources needed to maintain this testing site design might limit its ability to be implemented in resource-limited environments. The need for culturally tailored testing programs will continue even as vaccination programs are enacted across the country. Currently, reimbursement-based and traditional medical service delivery models often operate at a financial loss; greater public funding support is needed to sustain culturally tailored, low-barrier testing models that address ethnic and racial disparities in SARS-CoV-2 infection.
